# Shifting uncertainty intolerance: methylphenidate and attention-deficit hyperactivity disorder

**DOI:** 10.1038/s41398-020-01118-4

**Published:** 2021-01-06

**Authors:** Alekhya Mandali, Arjun Sethi, Mara Cercignani, Neil A. Harrison, Valerie Voon

**Affiliations:** 1 Department of Psychiatry, University of Cambridge, Cambridge, UK; 2 Institute of Psychiatry, Psychology & Neuroscience, Kings College London, London, UK; 3 Clinical Imaging Sciences Centre, Brighton and Sussex Medical School, University of Sussex, Brighton, UK; 4 Cardiff University Brain Research Imaging Centre, Cardiff University, Cardiff, UK

**Keywords:** Predictive markers, Human behaviour, ADHD

## Abstract

Risk evaluation is a critical component of decision making. Risk tolerance is relevant in both daily decisions and pathological disorders such as attention-deficit hyperactivity disorder (ADHD), where impulsivity is a cardinal symptom. Methylphenidate, a commonly prescribed drug in ADHD, improves attention but has mixed reports on risk-based decision making. Using a double-blinded placebo protocol, we studied the risk attitudes of ADHD patients and age-matched healthy volunteers while performing the 2-step sequential learning task and examined the effect of methylphenidate on their choices. We then applied a novel computational analysis using the hierarchical drift–diffusion model to extract parameters such as threshold (‘*a*’—amount of evidence accumulated before making a decision), drift rate (‘*v*’—information processing speed) and response bias (*‘z’ apriori* bias towards a specific choice) focusing specifically on risky choice preference. Critically, we show that ADHD patients on placebo have an *apriori* bias towards risky choices compared to controls. Furthermore, methylphenidate enhanced preference towards risky choices (higher *apriori* bias) in both groups but had a significantly greater effect in the patient population independent of clinical scores. Thus, methylphenidate appears to shift tolerance towards risky uncertain choices possibly mediated by prefrontal dopaminergic and noradrenergic modulation. We emphasise the utility of computational models in detecting underlying processes. Our findings have implications for subtle yet differential effects of methylphenidate on ADHD compared to healthy population.

## Introduction

Evaluation of risk is one of the core constructs critical to decision making. Risk-taking can involve the evaluation of explicit risk where the likelihoods of benefit (reward) or harm (loss) are known; or ambiguity, where the likelihoods are unknown^1^. Attention-deficit hyperactivity disorder (ADHD) is a neurodevelopmental disorder characterised by inattention, impulsivity, and decision-making deficits^2^. Higher risk-taking behaviours are a common feature of ADHD, which include substance abuse^3^ and gambling behaviours^4^.

A meta-regression analysis comparing multiple studies on risk-taking behaviour including multiple implicit (Iowa Gambling Task (IGT), Balloon Analogue risk task (BART)) and explicit forms (Game of dice, Card Playing Task, Cambridge Gambling Task (CGT) & Probabilistic discounting task)) showed greater risk-taking in ADHD^5^. Methylphenidate (MPH), a commonly prescribed medication has a differential influence on ADHD patients relative to healthy controls based on the sub-type of impulsivity (Table 1). These mixed differential effects call out for novel approaches to understand mechanistic differences and identify behavioural markers associated with the pathophysiology.

**Table 1:** Effect of methylphenidate on impulsivity subtypes in healthy controls and attention-deficit hyperactivity disorder.

Dimension	Effect on controls	Effect on ADHD
Response Inhibition	Enhanced correct Go responses^39,40^ associated with fronto-parietal activity^40^ in a Go-Nogo task, Improved response inhibition in a modified stop signal task^41^	Improved response inhibition on stop signal task to that of non-medicated controls^13,42^
Delayed reward	Decreased delay discounting^23^	Decreased delayed discounting in ADHD children^24^
Risk-taking	No difference in number of gambles or response time in controls^43^. Increase in gambles despite an increase in loss^38^	Mixed results in betting behaviour with both decreased^5^ and no effect reported^5,44^. Improved IGT score in inattention sub-type^44^
Reflection Impulsivity	No effect on Information Sampling Task in both controls and ADHD^5^. In controls, MPH differentially modulated the performance, depending on baseline impulsivity^45^	
Waiting Impulsivity	Increased premature responding in a 4 choice serial time task in controls^45^	

Recently, computational psychiatry has sought to explain how these behaviours are underpinned by altered computational processes during decision making^6–8^. Drift–diffusion models (DDM) assess and extract parameters corresponding to the underlying neural processes of decision making, by posing it as an evidence accumulation problem^9^. The three main parameters i.e., decision threshold (*a*) or boundary separation, an index of the amount of evidence accumulated prior to a decision; drift rate (*v*), an index of the speed of evidence accumulation; and response bias (*z*), the *apriori* tendency or bias towards one choice or another, represent different processes in choice reaction time paradigms.

Computational modelling in ADHD using DDM predominantly focused on either understanding the relationship between its parameters with proposed pathophysiological mechanisms such as reduced glutamatergic cortical drive; dopamine transfer deficit theory (or the deficit in transfer of dopaminergic responses to unconditioned rewards) and moderate brain arousal theory^10^ or task-related attention deficits, such as drift rate being an index of attention rather than measure of information processing^10^. Here we focus on the concept of risk or uncertainty tolerance with higher real world and clinical implications.

In this study, we exploit the capacity of a hierarchical modelling framework to decode the neural mechanisms of decision making specific to risk attitudes. We emphasise the role of response bias (*z*) currently underexplored in the ADHD literature, in risk-based decision making. Response bias is suggested to reflect the *‘apriori’* bias of an individual towards a specific choice, correlating with activity of fronto-parietal and fronto-basal ganglia networks^11,12^. Using hierarchical drift–diffusion modelling (HDDM), we previously analysed the two-step sequential learning task in compulsive disorder patients (obsessive compulsive disorder and alcohol dependence), in the context of uncertainty and conflict irrespective of choice preference^7^. Using the same task, we now apply a novel analysis, focusing on choice preference (risk attitudes) in ADHD and Healthy volunteers (HV) and compare the effects of MPH on each of the groups.

## Methods

### Participants and Protocol

A total of 49 participants (25-ADHD patients and 24-HV, a standard for behavioural studies with medication challenges between groups^8,13,14^) were recruited from a specialist clinic at Sussex Partnership NHS Foundation Trust and HV via classified advertisements and university mailing lists. The patient assessment included semi-structured interviews using the Diagnostic Interview for ADHD in adults (DIVA), completion of the Conner’s self-report Adult ADHD questionnaire, and review of school reports wherever possible. All patients had confirmed DSM-IV diagnoses of ADHD (See^8^ for complete details of selection/exclusion criteria). Local and national ethical approvals were obtained from Brighton and Sussex Medical School (14/014/HAR; 12/131/HAR) and the East of England (Hertfordshire) National Research Ethics Committee (reference: 12/EE/0256) and written informed consent was obtained. The demographics and clinical measures are reported in Table 2.

**Table 2:** Lists the (mean ± standard deviation) of the demographics, clinical measures, behavioural measures and the reinforcement learning estimates between attention-deficit hyperactivity disorder group and Healthy Controls.

Parameter	ADHD	HV	*p*-value	
Age	33.48 ± 9.8	31.04 ± 8.24	*ns*	
FSIQ	108.77 ± 6.63	108.85 ± 6.83	*ns*	
BDI	14.36 ± 8.7	6.63 ± 6.9	*0.001*	
ADHD Index	24.16 ± 5.72	8.96 ± 5.03	*<0.001*	
STAI Trait	54.28 ± 11.44	37.7 ± 10.88	*<0.001*	
Medication duration (months)	32 ± 40.8	Not applicable		
DSM—Inattentive	18.96 ± 4.8	7.16 ± 4.4	*<0.001*	
DSM—hyperactivity/Impulsivity	18.04 ± 6	4.7 ± 3.3	*<0.001*	
Gender—Male (Female)	15 (10)	16 (8)	*Χ*^*2*^*(1) = 0.234, p* = *ns*	

Behvaioural measures and Reinforcement Learning estimates				
Measure	Controls-Placebo	Controls-MPH	ADHD-Placebo	ADHD-MPH
Percentage of risky choices	0.52 ± 0.06	0.53 ± 0.06	0.53 ± 0.06	0.54 ± 0.07
Response time of risky choice (*ms*)	841 ± 163.7	804.2 ± 121	840 ± 222.5	833.1 ± 220.6
Response time of Non-risky choice (*ms*)	822.4 ± 147.6	800.7 ± 137.1	830 ± 214	840 ± 222.5
β_1_-Randomness Stage1	4.1 ± 3.7	5.7 ± 4.01	4.3 ± 2.6	4.45 ± 3.1
β_2_-Randomness Stage2	3.2 ± 1.68	3.7 ± 1.92	2.7 ± 1.76	3.48 ± 2.13
η_1_-Learning rate Stage1	0.52 ± 0.3	0.52 ± 0.27	0.46 ± 0.33	0.44 ± 0.35
η_2_-Learning rate Stage2	0.41 ± 0.25	0.43 ± 0.28	0.45 ± 0.31	0.42 ± 0.25
*ps*-Perseveration	0.13 ± 0.2	0.14 ± 0.17	0.1 ± 0.1	0.14 ± 0.18
*w-*Model free - model based	0.36 ± 0.26	0.36 ± 0.29	0.29 ± 0.23	0.24 ± 0.22

*MPH* methylphenidate, *ADHD* attention-deficit hyperactivity disorder, *HV* healthy volunteers, *FSIQ* Full Scale Intelligence Quotient, ADHD Index, *BDI* Beck Depression Inventory, *STAI* State-Trait Anxiety Inventory measures of healthy controls and patient group.

Exclusion criteria included any neurological or psychiatric history past or current, except for anxiety and/or unipolar depressive disorder currently in remission, history of significant head injury, and current drug or alcohol abuse. Each patient was managed on a stable regimen of methylphenidate (minimum 18 mg) or dexamphetamine (minimum 10 mg) for at least 2 months before study enrolment. For healthy controls, a history of serious cardiovascular conditions including cardiomyopathy, coronary artery disease, ventricular arrhythmia or hypertension, heart failure, current or recent use of monoamine oxidase inhibitors, anticoagulants, anticonvulsants or antipsychotics or a diagnosis of glaucoma were on exclusion criteria. Moreover, ADHD participants were routinely screened for these potential contra-indications to stimulant medication at clinical assessment.

The experimental design was a double-blinded placebo-controlled study with all participants completing two sessions of testing separated by a minimum of 1 week. ADHD patients were required to refrain from their regular medication on the test as well as 2 days before the day of testing. A.S who conducted the experiment and the entire participant testing was blind to treatment allocation. N.A.H (a qualified doctor) was aware of treatment allocation for safety reasons but was not involved in actual testing. During the first session, the participant in a randomised manner received either MPH or placebo in sealed envelopes. The alternate treatment was allocated in the second session. Patients with ADHD were given their normal morning medication dose or an inactive placebo whereas HV received 20 mg of MPH or placebo.

### The sequential learning task

**Fig. 1:**
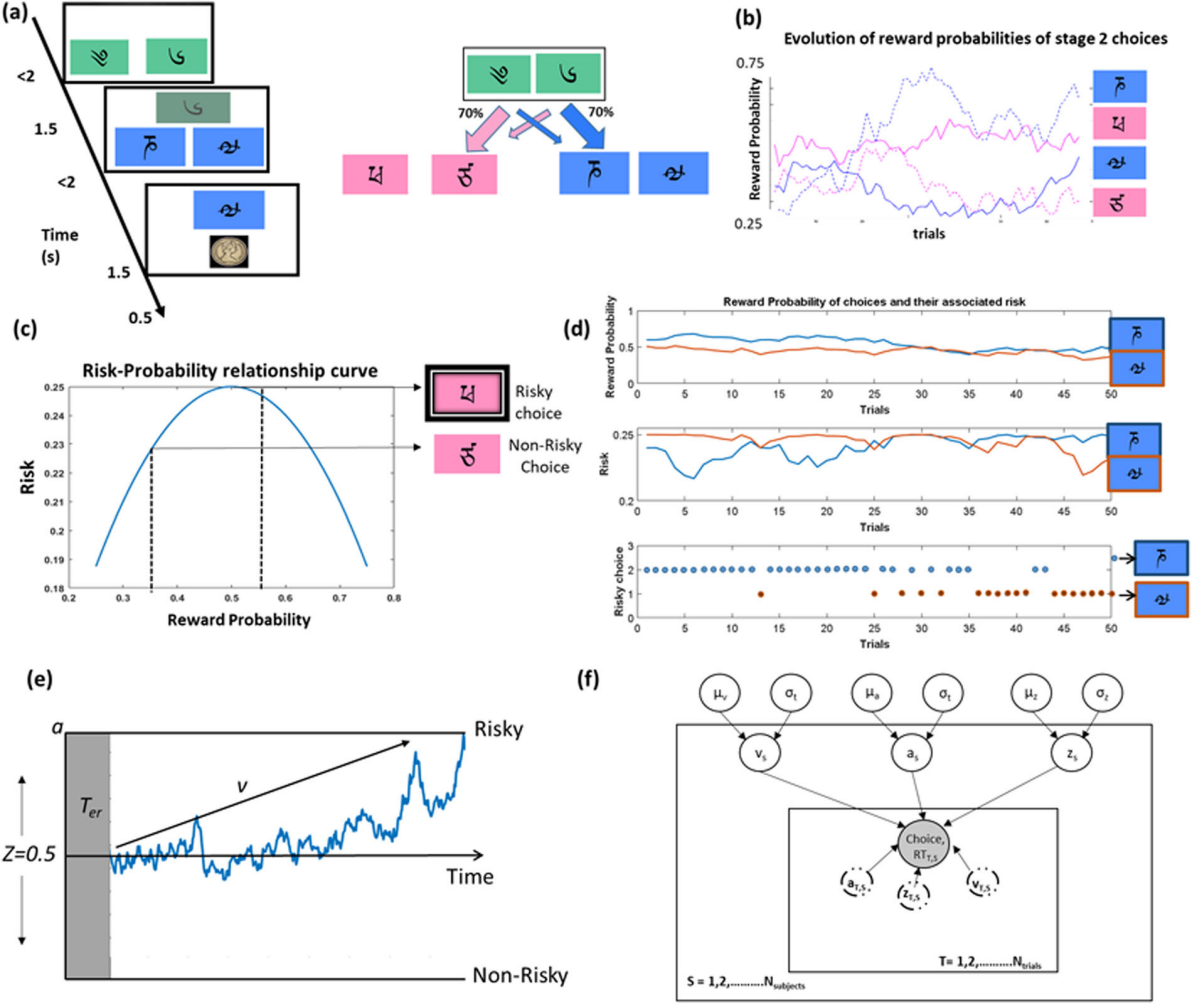
Sequential learning task, risk-probability formulation and hierarchical drift–diffusion model(HDDM) (**a**) 2-step sequential learning task (**b**) Evolution of all stage 2 choice reward probabilities across trials (**c**) risk/uncertainty as a function of second stage reward probability (Eq. 1) which follows an inverted U-curve (**d**) An example plot with reward probabilities of two stage2 choices, their corresponding risk(Eq. 1) and trial-wise risky choice among the two choices (**e**) pictorial representation of the drift–diffusion model with its parameters (threshold—*a*, drift rate-*v* and response bias-*z*) and (**f**) pictorial representation of the HDDM structure with input variables and estimates.

The widely tested and published sequential learning task^7,15,16^ consists of two stages; In stage 1, participants are presented with a stimulus pair (Fig. 1a). Upon selection, depending on the participant’s choice and stage 1 transition probability (*P* = 0.70 or 0.30), the second stage stimuli pair were then presented. The choice of a stimulus at stage 2 led to a reward (£1 or no reward). The reward probabilities of stage 2 stimuli were dynamic, based on a slow random Gaussian walk (*P* = 0.2–0.75, Fig. 1b). Subjects were allowed a decision time of 2 s at each stage, a 1.5 s transition time between stages, and observed the outcome for 1 s. Participants underwent a computerised self-paced training lasting 15–20 min and completed all 134 trials.

### Risk in the sequential learning task

In this study, the risk associated with a stage 2 choice was calculated based on their variance in reward probability (Fig. 1c, calculated using Eq. 1)^17^. This formulation comes from a theoretical framework of risk/uncertainty^17^ supported by experimental human imaging studies, where dopaminergic, striatal, and midbrain neural activity resemble the inverted U-curve while coding for risk^17,18^. This definition also controls for the confounding effect of the expected value of the choice from risk.

1 $$R_{x,j}^{i} = P_{x,j}^{i} ( {1 - P_{x,j}^{i}}),$$

where $R_{x,j}^i$is the risk variable, $P_{x,j}^i$is the reward probability of the transitioned stimulus ‘*x*’ at stage 2 for trial *i* and subject *j*. The riskiest choice was the stimulus whose reward probability was at chance level (*P* = 0.5), i.e., associated with the greatest variance in outcomes and the least risky choice was associated with either *P* = 0.25 or P = 0.75 with a greater likelihood of either winning or not winning and hence greater certainty (or lower uncertainty).

Using Eq. 1, we calculated the risk associated with the two choices (Fig. 1c) for each trial and labelled the option with the highest variance as the risky one (Fig. 1d). This information was then compared with the subject’s actual choice in a given trial, to classify it as either a risky or a non-risky choice. This was repeated for all the trials and subjects.

### Hierarchical drift–diffusion model (HDDM)

HDDM falls under the class of sequential sampling methods which utilise Bayesian methods to estimate the DDM parameters such as the threshold (*a*) and the drift rate (*v*), response bias (*z*), and non-decision time (*t*) (Fig. 1e, f). We focus our analysis on the first three parameters as *t* primarily concerns motor and non-decision-making processes. The Bayesian-based HDDM estimates parameters as posterior probability distributions with the mean of the distribution representing the group’s average. The model utilises the Markov Chain Monte Carlo sampling method to estimate the distributions. The prior distribution for each parameter was based on 23 studies that reported the best fitting DDM parameters for multiple cognitive tasks^19^. The pre-analysis code was written in MATLAB version 2017a and the built-in HDDM python package by^19^ was used for parameter estimation.

Trials with response times less than 50 ms were discarded from the analysis to ensure model convergence and to constrain the data to realistic response times. The parameters were estimated by drawing 120,000 samples with the first 10,000 samples being discarded as burn-in and saving only every 10^th^ sample. The convergence of the model was assessed by both visual inspection and computation of the Gelman-Rubin statistic, which indicated convergence (*R*^*^*^ < 1.1)^20^.

Additionally, we also estimated the parameters for accuracy (see^7^ for details on methods). We estimated all the three HDDM parameters (*a*, *v*, and *z*) in HV and ADHD in placebo and MPH conditions.

### Statistical Analyses

In line with the HDDM estimation of parameters, we used Bayesian methods as implemented in JASP for statistical analysis. Bayesian repeated measures ANOVA was used to test the significance across groups, conditions, and their interactions and, if significant, post-hoc Bayesian paired, and independent *t*-tests were used to assess the mean difference. Evidence for hypothesis testing was inferred from the Bayes Factor (BF10), with a BF10 > 3 indicating moderate evidence and >100 strong evidence in support of the alternate hypothesis^20^. The Bayes Factor used to report the evidence for (or against) a hypothesis was obtained from JASP. Based on JASP guidelines, the normality and homogeneity of variance was met by the HDDM estimates. The behavioural (response time and accuracy) and demographic measures were analysed using frequentist repeated measures ANOVA and post-hoc Bonferroni corrected independent paired and sample two-tailed *t* tests using SPSS version 25. The correlation was performed using Bayesian statistics.

## Results

### Behavioural measures—healthy volunteers and ADHD population

Repeated measures ANOVA showed no significant main effects of drug or group or their interaction in terms of response time or percentage of risky choices (See Table 2).

### Reinforcement learning parameters

Repeated measures ANOVA on the reinforcement learning (RL) parameters (beta (*β*_*1*_*, β*_*2*_*)*, learning rate (η_1_ and η_2_), and perseveration (*ps*), model free-model based weight (*w*)) of ADHD and HV subjects during placebo and MPH condition showed no main effect of drug or group or drug by group interaction. The individual estimates of the parameters are reported in Table 2.

To assess the effects of depression and anxiety on the behavioural measures and reinforcement learning estimates, we ran repeated measures ANOVA of reinforcement learning parameters during placebo and MPH condition with depression and anxiety scores as covariates. We observed no main effect of drug or depression or anxiety or any interaction effects. Similarly, repeated measures ANOVA with covariates on the behavioural outcomes (risk and reaction time) that form the primary inputs to the HDDM model also showed no main effect of drug or group or risk or effect when adjusted for depression and anxiety covariates.

### HDDM model estimates of Healthy volunteers and ADHD population

Using the extracted choice (risky vs non-risky) and response time information (Fig. 1f), we estimated the parameters (*a, v* and *z*) for ADHD and HV population when on drug or placebo. At baseline on placebo, both ADHD and HV had a response bias (*z*) towards the more certain choice suggesting lower uncertainty tolerance.

Bayesian repeated measures ANOVA showed no evidence for the main effect of the drug, group, or its interaction for either threshold or drift rate.

**Fig. 2:**
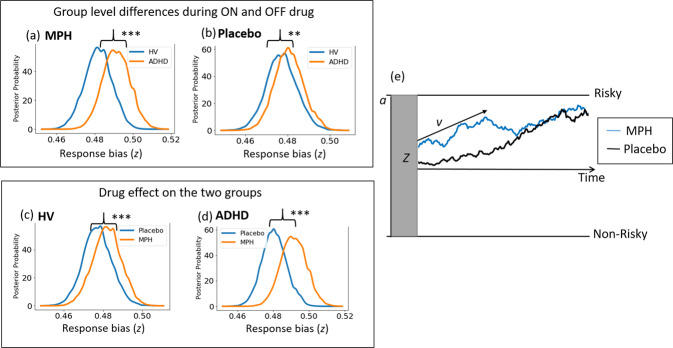
Effect of methylphenidate (MPH) on the starting or response bias in control and patient groups. (**a**, **b**) Comparison of response bias (*z*) in healthy volunteers (HV) and attention-deficit hyperactivity disorder (ADHD) on (MPH) and placebo (**c**) Comparison of response bias (*z*) of HV on MPH or placebo and (**d**) Comparison of response bias (*z*) of ADHD on MPH or placebo and (**e**) Pictorial representation of evidence accumulation process and the effect of MPH.

For response bias (*z*), repeated measures Bayesian ANOVA (Fig. 2) showed a very strong evidence for a main effect of drug (BF_10_ = 6.03 × 10^11^), group (BF_10_ = 86344) and group by drug interaction (BF_10_ = 3.65 × 10^6^). Post-hoc Bayesian independent sample t-tests showed very strong evidence for the patient group to have a higher preference towards the risky choice when on MPH relative to HV (BF_10_ = 8.94 × 10^14^) and strong evidence when on placebo relative to HV (BF_10_ = 20.9). To check the effect of drug individually on each of the groups, we also ran Bayesian paired sample t-tests and show strong evidence for the drug to induce a preference towards the risky choice particularly in ADHD (BF_10_ = 1.16 × 10^10^) but also in HV (BF_10_ = 397.1).

The inclusion of clinical scores such as depression (BDI), anxiety (STAI-T), Inattention (DSM-I) and hyperactivity (DSM-H) scores as covariates, did not alter the strong evidence for the main effect of drug (5.88 × 10^11^), group (BF_10_ = 88086) and their interaction (BF_10_ = 3.9 × 10^6^) without any covariates. There was no evidence for an effect of BDI score (BF_10_ = 2.7) but was observed for anxiety (BF_10_ = 36), DSM-I (BF_10_ = 138) and DSM-H (BF_10_ = 352). We further show evidence for an interaction effect between drug and anxiety (BF_10_ = 46), drug and DSM-I scores (BF_10_ = 1287) and drug and DSM-H scores (BF_10_ = 213) but not group interaction effects with depression or inattention or hyperactivity

**Fig. 3:**
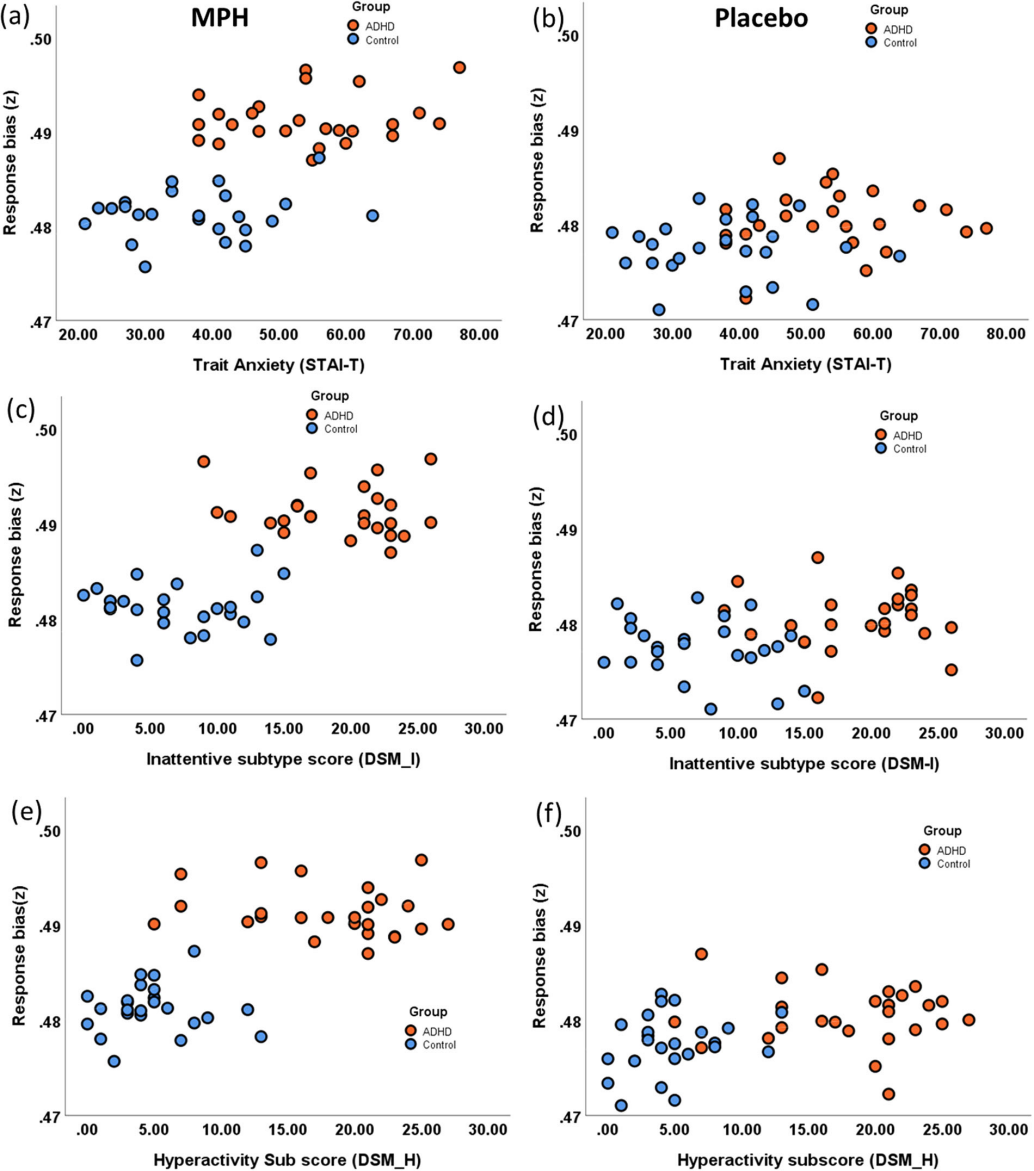
Relationship between clinical scores and the response bias (*z*) parameter of the hierarchical drift diffusion model. Shows the scatter plot between response bias (z) and anxiety, inattention and hyperactivity scores in methylphenidate and placebo conditions in patient (orange) and healthy control (blue) groups (**a**) and (**b**) show the correlation between the anxiety score (STAI-trait) and response bias in placebo and drug respectively. **c**, **d** correlation between response bias and clinical inattention sub-type score (DSM-I) and (**e**) and (**f**) correlation between response bias and clinical hyperactivity sub-type score (DSM-H) in placebo and drug conditions.

To further understand this interaction effect between drug and clinical scores (anxiety, DSM-I and DSM-H), we calculated Bayesian correlations within individual groups between response bias and clinical scores in drug and placebo conditions. Anxiety and ‘*z*’ were not correlated within each group (Fig. 3a, b) for drug (*r*_HV_ = 0.17, BF_10_ = 0.3; *r*_ADHD_ = 0.17, BF_10_ = 0.3) or placebo (*r*_HV_ = *−*0.01, BF_10_ = 0.25; *r*_ADHD_ = 0.08, BF_10_ = 0.3). Similarly, inattention and ‘z’ were not correlated within each group (Fig. 3c, d) for drug (*r*_HV_ = 0.03, BF_10_ = 0.26; *r*_ADHD_ = *−*0.13, BF_10_ = *0.3*) or placebo (*r*_HV_ = *−0.24*, BF_10_ = *0.47*; *r*_ADHD_ = .005, BF_10_ = 0.25). Hyperactivity and ‘z’ were also not correlated within each group (Fig. 3e, f) for drug (*r*_HV_ = 0.01, BF_10_ = 0.25; *r*_ADHD_ = −0.2, BF_10_ = 0.4) or placebo (*r*_HV_ = 0.26, BF_10_ = 0.52; *r*_ADHD_ = −0.09 BF_10_ = 0.27). Our findings suggest the drug alone influences response bias in ADHD subjects irrespective of clinical features.

To highlight the specificity of MPH on risky decision making, we also extracted the parameters (*a* and *v*) pertaining to accuracy (See^7^ for details). Repeated measures ANOVA did not show any evidence for the main effect of group or drug or their interaction for both thresholds as well as drift rate.

## Discussion

We show that ADHD patients when off medication have an *apriori* bias towards risky uncertain choices compared to controls. Methylphenidate enhanced preference towards risky choices (higher *apriori* bias) in both groups but had a significantly greater effect in the patient population. Thus, methylphenidate appears to shift tolerance towards risky uncertain choices possibly mediated by prefrontal dopaminergic and noradrenergic modulation. Using this novel application of HDDM to the sequential learning task, we focus on choice preference for risk and show the capacity to detect subtle differences, a unique strength of computational psychiatry relative to sole reliance on behavioural outcomes^6,7,21^.

We have previously dissociated contextual conflict and uncertainty with HDDM using this sequential learning task but did not take into account choice preference^7^. Here, we focused on choice preference, defining risk as greater outcome variance. This task captures an index of implicit risk as the probabilities are not explicitly known or defined and must be inferred or learned from outcomes. Although it might be tempting to interpret the findings as enhanced risk biases, they are consistent with a more adaptive or functional interpretation of MPH modulating uncertainty processing^22^ as the behavioural outputs remain unaltered.

Although ADHD has a higher response bias towards risky uncertain choice compared to healthy controls, at baseline both groups displayed greater underlying uncertainty intolerance with a bias towards the more certain choice. Uncertainty intolerance may be an important component of decisional impulsivity with choices made to avoid risky uncertain conditions. For instance, delay discounting may reflect intolerance of the uncertainty surrounding the likelihood of receiving the delayed choice or rapid decisions in the context of uncertainty or conflict that may similarly reflect discomfort with uncertainty. Thus, our results are consistent with results obtained from previous delay discounting studies, where both controls and ADHD participants chose higher delayed rewards in the presence of MPH^23,24^ possibly also indicating a higher tolerance towards uncertainty.

Our findings show a specific effect of methylphenidate on response bias, which reflects the priors or pre-existing expectations or biases towards the selected choice (here, the risky uncertain choice). This effect of methylphenidate on ADHD is unrelated to comorbid clinical features such as depression or anxiety or ADHD subtypes.

Mechanistically, the response bias parameter has been observed to correlate with the activity of fronto-parietal and fronto-basal ganglia networks^11,12^, areas dysfunctional in ADHD^2^. Within the fronto-parietal-basal ganglia circuitry, Lopez and colleagues observed the response bias parameter (estimated from a DDM) to correlate with the activity of ventromedial prefrontal cortical (vmPFC) during a multi-categorical value-based decision-making task^25^. The vmPFC, in addition to encoding value^26,27^, is implicated in risk-taking^26,28,29^, with structural abnormalities such as lower volumes, presence of stroke lesions and volume of damage in the medial orbito-frontal (mOFC) region of the vmPFC associated with increased risk-taking^30,31^. ADHD adolescents who are at high risk of developing substance abuse had higher vmPFC activation compared to age-matched controls while processing riskier outcomes^32^. Similar to the task used by Lopez and colleagues, the sequential learning task used in this study is also a value learning task, which requires the participants to learn the values (reward probabilities) of the choices and estimate the risk or uncertainty associated with them. Further imaging studies should focus on the vmPFC, which encodes both value and risk, as a potential anatomical candidate underlying the risk response bias parameter (*z*).

Mechanistically, MPH enhances the prefrontal dopaminergic and noradrenergic levels by inhibiting the DA and NE transporters and by indirectly activating D1-dopaminergic and α_2_-norepinephrine^33^. In ADHD patients, MPH enhanced the hypoactive vmPFC on placebo comparable to the observed levels of healthy controls^2^. Dopaminergic medications increase risk-taking by changing the subjective weighting of the reward probabilities^34^ or by increasing the baseline gambling tendency^35^. In this study, it is very likely to have similar effects where MPH via D1 and α_2_-norepinephrine receptors enhanced the vmPFC activity (one of brain’s core valuation system^36^) and altered the subjective value of the choices. This altered representation may then be reflected as a shift in the *‘apriori’* bias (response bias parameter) towards the risky choice. Further studies are required on risk representation to systematically understand the effects of MPH in ADHD subjects.

This study is not without its limitations. We have not systematically varied the dosage of MPH and assessed dose-related effects on risk-taking and response bias. The task primarily designed to be an implicit-value learning task rather than a risk evaluation task may influence the behaviour and the outcome.

To summarise, using a novel computational analysis on choice preference, we show that ADHD subjects have an apriori bias towards risk, enhanced with MPH compared to healthy controls. These findings might suggest a higher tolerance towards risk or uncertainty. Computational estimates may act as potential biomarkers capturing subtle effects of medication on risk behaviours not manifested in behaviours^7,36,37^. Our findings have relevance not just in ADHD but also in the impulsive-compulsive disorder spectrum, or the effects of cognitive enhancers. A risk-based study in Parkinson’s disease previously showed similar effects between MPH and pramipexole, a D2/D3 dopamine receptor antagonist prescribed for PD^38^. Critically we show using a single task commonly used to assess goal-directed and habit control, the capacity to assess both contextual uncertainty and conflict^7^ and risk uncertainty preferences. Our findings highlight the sensitivity of computational models to identify the subtle changes in disease and the effect of drug.

## Data Availability

The analysis code used to estimate the computational parameters will be made available on request.
